# Small is beautiful: In defense of the small-*N* design

**DOI:** 10.3758/s13423-018-1451-8

**Published:** 2018-03-19

**Authors:** Philip L. Smith, Daniel R. Little

**Affiliations:** The University of Melbourne, Melbourne, Australia

**Keywords:** Methodology, Replication, Inference, Mathematical psychology

## Abstract

The dominant paradigm for inference in psychology is a null-hypothesis significance testing one. Recently, the foundations of this paradigm have been shaken by several notable replication failures. One recommendation to remedy the replication crisis is to collect larger samples of participants. We argue that this recommendation misses a critical point, which is that increasing sample size will not remedy psychology’s lack of strong measurement, lack of strong theories and models, and lack of effective experimental control over error variance. In contrast, there is a long history of research in psychology employing small-*N* designs that treats the individual participant as the replication unit, which addresses each of these failings, and which produces results that are robust and readily replicated. We illustrate the properties of small-*N* and large-*N* designs using a simulated paradigm investigating the stage structure of response times. Our simulations highlight the high power and inferential validity of the small-*N* design, in contrast to the lower power and inferential indeterminacy of the large-*N* design. We argue that, if psychology is to be a mature quantitative science, then its primary theoretical aim should be to investigate systematic, functional relationships as they are manifested at the individual participant level and that, wherever possible, it should use methods that are optimized to identify relationships of this kind.

*“It is more useful to study one animal for 1000 hours than to study 1000 animals for one hour”* — B. F. Skinner (quoted in Kerlinger and Lee (1999))

Since the cognitive revolution of the 1960s, the dominant paradigm for inference from data in scientific psychology has been a null-hypothesis significance-testing one. According to this paradigm, the goal of experimentation is to draw inferences about the properties of an underlying population or populations from measurements made on samples drawn from those populations. Expressed in model-comparison terms (Maxwell & Delaney, 1990), the goal of inference is to decide between two models of the psychological phenomenon under investigation, or more precisely, to decide between two models of the *data-generating process* that gave rise to the observed experimental outcomes. One model is a null model, in which the value of a parameter in the data-generating process is zero; the other is an alternative model, in which the value of the parameter is nonzero. Subject to the assumption that the experimental units are random and a representative sample from some larger population—needed to ensure statistical unbiasedness—the estimate of the parameter of the data-generating process from the sample is used to infer the value of a hypothetical “population parameter,” which characterizes the experimental effect. This parameter is conceptualized as the numerical value that would be obtained if a population-wide census could feasibly be undertaken.

Recently, our faith in this inference paradigm has been severely tested. Psychology, we are told, is in the grip of a “replication crisis,” which threatens to shake the discipline to its epistemological foundations. The most persuasive evidence for a discipline in crisis comes from the Open Science Collaboration (OSC & et al. 2015; Aarts et al., 2015), which reported the results of 100 attempted replications of published studies, predominantly in social and cognitive psychology. Around two-thirds of the OSC’s attempted replications were unsuccessful and, when they were successful, the effect sizes were often smaller than those in the original studies. While the extent, magnitude, and causes of the putative replication crisis have been, and continue to be, disputed (Gilbert et al., 2016), the implications of the OSC’s findings have not been lost on either the scientific or the popular press. A commentary in *Nature* (Baker, 2015) asserted: “Don’t trust everything you read in the psychology literature. In fact, two-thirds of it should probably be distrusted.”

Faced with evidence of a crisis, various suggestions have been made for reforming experimental and data-analytic practices (Gelman & Loken, 2014; Gelman, 2015; Simmons et al., 2011). One suggestion is to change the criterion of “statistical significance” (Benjamin et al., 2017), which would change the decision rule for choosing between the null and the alternative model, raising the threshold for “discovery.” Another suggestion has been to advocate the use of much larger samples of participants, supported by formal power calculations. Indeed, lowering the criterion for statistical significance (e.g., to the *p* < .005 as suggested by Benjamin et al., (2017)) would necessitate a 70% increase in the sample size to achieve 80% power (Lakens et al., 2017). Further, some leading journals that publish cognitive research have begun automatically to decline work that uses small samples. The stated reason for doing so is the “unreliability” of inferences based on small samples: Standard errors of estimate of population parameters based on small samples are large, so the resulting scientific inferences—it is reasoned—must be inherently untrustworthy. This editorial stance reaffirms the view that the ultimate goal of data analysis is to estimate population parameters from measures aggregated across the individuals in a sample.

Our goal in this article is to argue for a contrary view. We argue that some of the most robust, valuable, and enduring findings in psychology were obtained, not using statistical inference on large samples, but using small-*N* designs in which a large number of observations are made on a relatively small number of experimental participants. We argue that, if psychology is to be a mature quantitative science, its primary theoretical aim should be to investigate systematic, functional relationships as they are manifested at the individual participant level. The estimation of population parameters, while not unimportant, is arguably of secondary concern and should probably be investigated using more refined techniques for characterizing individual differences than the blunt instrument of simple averaging that conventional statistical methods provide. For one, averaging tends to obscure important qualitative individual differences (see, for example, Estes and Maddox (2005), Liew et al., (2016), and Navarro et al., (2006)). More importantly, in most of the cases in which the replicability of psychological data has been called into question, the populations to which the inferences were intended to apply were never specified in any demographically precise way because they were never of central theoretical importance to begin with. Such practices beg the question: What does it mean to estimate a population parameter when the defining features of that population are specified only imprecisely? Further, how can samples which are inherently biased toward certain demographic groups (Henrich et al., 2010) be expected to provide any strong claim toward generality? Our ultimate aim is to assert the validity and legitimacy of the small-*N* design as a method for generating reliable psychological knowledge and to rescue it from what we see as a rising tide of “large samples good, small samples bad” thinking that has threatened to swamp the recent methodology debate.

To foreshadow the contents of the rest of the article, our primary aim is not to denigrate current research practices, but, rather, to provide a sharper and more balanced appraisal of what we believe are the often-overlooked merits of small-*N* designs. These designs concentrate their experimental power at the individual participant level and provide high-powered tests of effects at that level. As a result, they are in a sense automatically “self-replicating” (Little & Smith, 2018), as we discuss more fully later. In addition to their self-replicating properties, small-*N* studies often embody a number of hallmarks of good scientific practice, particularly as they pertain to precise measurement, effective experimental control, and quantitatively exact theory. Although there is no necessary connection between the use of small-*N* designs and these other features of scientific practice, many researchers who engage in highly quantitative psychological science often favor small-*N* designs because they see them as possessing distinct advantages. Part of our aim is to argue that the focus on sample size as the sole or even the primary cause of unreliable psychological knowledge is to lose sight of much of what makes good science. To us, it is a source of irony that, in the current climate of uncertainty and methodological re-evaluation, studies that embody what we believe are characteristics of good science can be rejected by journal editors as a priori “unreliable.” We therefore wish to challenge the reductive view that the only route to reliable psychological knowledge is via large samples of participants. Parenthetically, we note that while we frequently contrast small-*N* and large-*N* designs for expository purposes we really view them as ends of a continuum and, for many researchers, the methodological sweet spot may lie somewhere in between (see e.g., Rouder and Haaf (in press)). We touch on this issue later.

Recent articles by Grice et al., (2017) and Normand (2016) make similar points to those we make here. Like us, Normand stresses the importance of repeated measures on single individuals as a reliable and in many ways, preferable, approach for understanding psychological phenomena. Grice et al. highlight the errors in inference that can arise when individual-level hypotheses are tested at the group level, especially when the population to which the group belongs is not clearly specified. Our critique also recalls elements of the now-classic papers by Meehl (1967, 1990), particularly in our distinction between process-oriented and phenomenon-oriented research and our emphasis on parameter estimation and prediction as an alternative to significance testing.

Our view is that the emphasis on statistical inference and the need to reform statistical practice as a response to the replication crisis has led us as a discipline to focus unduly on one part of the totality of scientific practice to the exclusion of others. That we have done so is probably because of psychology’s excessive reliance on statistical inference as a way to do science. We believe that this reliance on statistics has become a kind of crutch that often leads us to neglect other elements that are crucial to good science. Among psychology’s endemic methodological problems, which are only indirectly related to statistical inference, are:

1. *Weak measurement.* Many psychological phenomena are characterized using measurement instruments whose relationship to the theoretical entities researchers wish to investigate is unknown, but which is likely to be at best, in S. S. Stevens’ (1951) typology, ordinal. This means that an unknown monotonic transformation intervenes between the latent construct of interest and its measured expression.
2. *Weak theories and models.* Many theories, and their formal expression in psychological models, are weak in either or both of two different ways. First, their predictions are often only ordinal: “If such-and-such a condition holds, then performance in Condition *A* is expected to be greater (faster, more accurate, etc) than in Condition *B*.” These kinds of weak ordinal predictions contrast sharply with the strong functional forms that characterize predictive relationships in other parts of science. Second, the predictions are often *sparse*. In some cases, theoretical inferences are drawn on the basis of a single point estimate or a comparison of a pair of point estimates (i.e., the textbook *t*-test). The sparseness of the empirical predictions can be quite unrelated to the internal complexity of the theories themselves. In the early days of connectionism, for example, many published articles attempted to draw inferences about the properties of the neural networks supporting particular cognitive functions from ordinal comparisons of simulated network performance and experimental data in a small number of conditions (Seidenberg & MClelland, 1989). The weak measurement problem and the weak theory problem are intimately connected: If measurement is only ordinal then theoretical predictions can never be stronger than ordinal.
3. *Poor experimental control of error variance.* The methodological gold standard in many areas is one in which a group of naive participants serves in a single experimental session and are all exposed to exactly the same experiment treatment. Any variability among participants in how they respond to the treatment is conceptualized as “experimental error.” Such error, and the fact that it is often large, is usually accepted as one of the inescapable realities of psychological experimentation. Because statistical power is inversely related to error variance, the only recourse when confronted with large error variance is to increase sample size. So ingrained has this methodology become in our way of doing science that it is easy to forget that there are other ways of tackling the problem of error variance. To do so requires us to ask: Why are people variable on this task and are there ways to reduce this variability?^1^

We discuss some of the implications of Points 1 to 3 in the following sections.

## What should we expect to find in a small-*N* design?

The proposition that small samples lead to unreliable, hard-to-replicate findings leads to the expectation that the replication crisis should be deepest in those areas in which samples are habitually the smallest, like the sensory sciences and animal learning studies. And we might expect that the crisis would have spilled over into other areas that have adopted the small-*N* approach, such as the cognitive neuroscience of awake, behaving animals. Indeed, by this reasoning, the very worst—the most methodologically irredeemable, epistemologically beyond-the-pale cases—should be those studies in which the research was carried out on only a single participant! However, two of the foundation stones of cognitive psychology, Weber’s Law, along with Fechner’s generalization of it Link (1992), and Ebbinghaus’s Law of Forgetting (Baddeley, 1997), were both carried out in this way: by a single experimenter (wholly, in the case of Ebbinghaus, and largely in the case of Fechner) administering a vast number of experimental trials to himself. As well as involving only single participant, there can be no suggestion as to the participant’s naivety in either of these cases. Despite flouting some of the most sacred methodological canons, Fechner’s and Ebbinghaus’s results have stood up robustly for more than a century. They are fixtures in textbooks; they are among the comparatively few results in psychology that we dignify with the name of “law”, and they stand as yet unbuffeted by the epistemological winds of the replication crisis.

In making these claims for findings like Fechher’s and Ebbinghaus’s laws, we are of course not attempting to suggest that all of the historical studies carried out using small-*N* or single-participant designs yielded enduring and reliable knowledge. That would be like claiming that all the best songs were written in the sixties because the songs from the sixties that continue to be played today are better than the songs being written now—and would embody the same kind of logical error. Rather, it is to claim that these examples show that use of single-participant designs is in no way inimical to the discovery of precise quantitative relationships of enduring psychological significance. Indeed, it might have been much more difficult for Fechner and Ebbinghaus to have discovered their laws had they worked with large-*N* designs. Moreover, the most convincing way to investigate these laws today continues to be at the individual level.

In a recent survey of the role of small-*N* designs in psychology, Saville and Buskist (2003) pointed out that the use of large-*N* designs is a comparatively recent phenomenon, which they linked to the publication of R. A. Fisher’s *Statistical methods for research workers* in 1925 (Fisher, 1925). They noted that the foundational studies of animal learning by Pavlov and Thorndike, some decades before Fisher’s book, were carried using small samples and that this practice continued in the work of B. F. Skinner (1938)—who provided us with the epigraph for this article—and his contemporaries, and that the small-*N* design remains the mainstay of animal learning studies to this day. They also noted that the historical shift from small-*N* to large-*N* designs was traced by Boring (1954). He analyzed the studies published in the *Journal of Experimental Psychology* in the years 1916, 1933, and 1951 and reported that in 1916 not a single study used a large-sample design, but by 1951 the number had risen to over 50%. Until the current replication crisis, this history might reasonably have been read as one of psychology gradually learning to use more appropriate and reliable methods—a process that was largely complete by the time of the cognitive revolution of the 1960s. As a corollary to this interpretation, we might expect that the areas of psychology that were slowest in taking advantage of Fisher’s methodological innovations would be those in which the replication crisis is now deepest. But this is not what we seem to find.

We take vision science as an example, as the sensory science nearest to the areas of cognitive and mathematical psychology in which we as authors work. We could have made similar points with reference to other sensory sciences, like auditory science, or animal learning. Among the primary outlets for research in vision are journals like *Vision Research,Journal of the Optical Society of America, A*, and more recently, *Journal of Vision*, in which the small-*N* design has tended to be the dominant paradigm. Published studies in these journals often report data from as few as two or three participants (typically styled “observers”), often members of the laboratory, and consequently far from naive. Our reading is that vision science shows no evidence of being in the grip of the kind of replication crisis that is currently afflicting cognitive and social psychology. Although there are unsolved problems, unanswered questions, and ongoing controversies in this area as in any other, vision science provides us, overall, with a highly coherent picture of how stimuli are coded and represented in the visual system. Vision science has undoubtedly benefited from the close theoretical link between behavior and physiology; but even with this qualification, there seems to be no evidence that its habitual use of small samples of participants has led to a replication crisis of a particularly virulent kind.

So what, then, is different about vision science? We quote, verbatim, from one eminent vision scientist, John Ross (2009, pp. 245-246), whose remarks are illuminating:

Research in visual perception began to make increasing use of measurement techniques developed for the study of psychophysics, and to adapt these for its own purposes. [...] Psychophysical techniques have provided visual science with something that is rare in psychology: measurements that are precise and replicable, on ratio scales such as area of time, or even on dimensionless scales, such as contrast or sensitivity. Because these measures are precise and replicable, and because, when they are applied, individual differences between observers tend to be small, papers reporting careful measurements on afew individuals or even asingle person are now perhaps more the rule than the exception, in the perception literature. Such measurements freed vision scientists from the inconvenience of having to use large numbers of participants and from the necessity to assess effect sizes in terms of individual variation. The use of precise models to explain, or even better, quantitatively predict the results of experiments became common.”

Ross’s remarks touch on all three areas of methodological weakness we identified above. We comment on each in turn.

1. *Strong measurement.* Ordinal-level hypothesizing is relatively rare in vision science because many experiments are concerned with characterizing visual sensitivity to stimuli that are either defined on ratio scales or are dimensionless transformations of ratio scales. Typical variables of interest in vision science include exposure duration, luminance contrast, chromatic contrast, summation area, summation time, motion direction, motion speed, motion coherence, spatial frequency, and orientation. These quantities are usually measured via individual *thresholds*: that is, the value of a ratio-scale or dimensionless variable that produces a criterion level of performance (often taken as the midpoint between floor and ceiling) in an individual participant making a perceptual judgment. Although such measurements presuppose a continuous, invertible, nonlinear mapping from the stimulus to the observed performance, the mapping itself (the *psychometric function*) is theoretically well specified and empirically testable.
2. *Strong theories.* Unlike many of the results in the OSC’s replication study, vision science has benefited from the widespread use of strong quantitative models. This practice dates from the introduction of linear systems theory into vision science by de Lange (1952, 1954, 1958) to characterize visual temporal sensitivity. The idea promoted by de Lange, that visual mechanisms can be characterized theoretically and mathematically as filters possessing specific spatiotemporal response properties, which possess the linear-system property of superposition, has proven to be an enormously fruitful one (Robson, 1966; Sperling & Sondhi, 1968; Watson, 1986), and one which has allowed researchers to make systematic theoretical connections between behavior and physiology. The behavioral link is typically made via detection-theoretic concepts that predict a relationship between the response of a visual mechanism to a given stimulus and the observer’s psychophysical judgment about it Graham (1989), where the latter may be expressed variously as a threshold, a choice probability, or a sensitivity index.
  One the implications of the use of strong quantitative models is that the research focus changes from significance testing to model fitting. The primary question is no longer “does performance in these two conditions differ significantly?” but “does my model agree with my data within the limits of my measurement precision?” Because the dominant focus is changed from inference to measurement, this kind of reasoning might (slightly provocatively) be termed “Gaussian,” to distinguish it from either classical or Bayesian inference and because it is reminiscent of Gauss’s use of the concept of “probable error” to characterize discrepancies between theory and empirical measurements (Sheynin, 1979). In psychology, these kinds of question are best asked at the individual observer level, where measurement precision is controlled by the number of trials performed by the observer in each stimulus condition. The goal of maximizing measurement precision naturally leads to designs in which a small number of observers perform a large number of number of experimental trials, rather than the converse, and to methods that minimize within-condition, within-observer variance. Because models in vision science typically predict performance across a range of stimulus and performance levels simultaneously—that is, across the entire psychometric function (Lu & Dosher, 1999)—these kinds of models avoid the sparse prediction problem and the associated reliance on significance testing of point hypotheses.^2^
3. *Effective control of error variance.* Two practices commonly used in vision science to control error variance have meant researchers have not been prey to the variation usually found in groups of naive observers. One is the use of practiced, experienced observers; the other is the use of stimulus manipulations designed to put all observers at a particular, criterion level of performance in a baseline or reference condition. Researchers who do small-*N* studies would agree that people are most variable, both in relation to themselves and in comparison to others, when they begin a task, and that within-observer and between-observer variability both decrease progressively with increasing time on task. While the nature of the processes by which people’s performance changes over time—the process of *perceptual learning*—is an important question in its own right (Dosher & Lu, 1999), in many studies the primary goal of research is to characterize the resolution, sensitivity, or capacity limits of the system. These questions are best asked in relation to practiced rather than naive observers in which the system is performing at its theoretical limits under optimum conditions.
  The second practice is the use of stimulus manipulations designed to equate performance across observers by putting them all at the same operating point. If, for example, stimulus contrast is thought to be the relevant variable, then a frequent practice is to scale the stimulus contrasts for individual observers to be multiples of their detection or discrimination thresholds for the task. If the experimenter’s hunch is correct and contrast is indeed the relevant variable for performance, then scaling contrast levels individually to make stimuli functionally rather than physically equivalent can produce a marked reduction in inter-observer variance. The combination of practiced observers and individually tailored stimulus manipulations has meant, as J. Ross (2009) pointed out, that there is high degree of inter-observer agreement in many tasks, obviating the researcher from the need to run large numbers of observers.

## Psychophysical methods in cognitive and mathematical psychology

The use of experimental methods characterized by J. Ross (2009) as “psychophysical” are not confined to vision and other sensory sciences, but are also common in cognitive psychology, especially in those areas of cognitive psychology that use explicit mathematical models. Among these areas are categorization (Ashby & Alfonso-Reese, 1995; Fifić et al., 2010; Nosofsky, 1986; Nosofsky & Palmeri, 1997), recognition (Nosofsky et al., 2011; Osth & Dennis, 2015; Raaijmakers & Shiffrin, 1981; Ratcliff, 1978; Shiffrin & Steyvers, 1997), decision-making (Busemeyer, 1985; Busemeyer & Townsend, 1993; Roe et al., 2001), working memory (Cowan, 1999), episodic memory (Dennis & Humphreys, 2001), psychophysical studies of attention (Bundesen, 1990; Smith, 1995; Smith & Ratcliff, 2009), and systems factorial technology studies of cognitive architecture (Townsend & Nozawa, 1995; Little et al., 2017). This list is not in any way intended to be exhaustive and simply reflects some of the areas in which we as researchers are personally interested. The common aim of experimental design in all of these areas is to try to maximize the contact between theory and data. The scientific intuition behind this design imperative is that a theory or model that predicts performance in many conditions simultaneously is more persuasive than one that predicts performance in relatively few conditions. For example, models of speeded decision-making like the diffusion model (Ratcliff, 1978) and other similar models (Brown & Heathcote, 2008; Usher & McClelland, 2001) predict entire families of response times distributions for correct responses and errors and the associated choice probabilities and how these jointly vary as a function of experimental conditions. This unusually rich contact between theory and data allows for very stringent model tests. To take another example, models of categorization like the generalized context model (Nosofsky 1984, 1986) or decision-bound models (Ashby & Gott, 1988; Ashby & Lee, 1991) predict observers’ categorization performance from their identification performance, not merely on a single set of categories, but across a variety of different category structures simultaneously at the level of individual items. The theoretical power and psychological interest of these models comes from their rich and detailed contact with data across multiple experimental conditions. Similar points could be made about experimental design and model testing in any of the other areas we have listed.

What these areas have in common is that theory testing is carried out by fitting (or otherwise evaluating) explicit mathematical models and that the relevant model properties are most directly expressed at the individual participant level. Of course, this kind of research is only possible with experimental manipulations that do not produce strong carry-over effects (Keppel 1982, p. 371), because model-testing at the individual level requires a sufficient number of experimental trials to produce an adequate level of within-condition measurement precision. The requirement that experimental manipulations not produce strong carry-over effects is needed in order to allow consecutive trials to be treated as statistically independent. In practice, because of the sequential effects that are found in many cognitive tasks (Luce, 1986; Treisman & Williams, 1984), models that assume independent trials can only approximate the true probability structure of such tasks. Sequential effects complicate the task of defining an appropriate goodness-of-fit measure for model evaluation, but the problems are not insurmountable. One way of dealing with the effects of uncontrolled (i.e., unmodeled) sources of across-trial variability in probability models is to use an appropriate model of statistical *overdispersion* (McCullagh & Nelder, 1989; Smith, 1998). Another way is to model the sequential dependencies directly (Jones, Curran, Mozer & Wilder, 2013; Little et al., 2016).

The fact that mathematical models most often predict performance at the individual participant level does not automatically preclude aggregation, by averaging or some other means, across participants, but does imply that conclusions made at the group level ought to be verified at the individual participant level (Grice et al., 2017). This implies that a sufficient number of trials must be collected at the individual level in order for such verification to be meaningful. Whether averaging or aggregation leads to artifacts that can distort inference depends wholly on the domain and the models in question and it is difficult to give a priori, domain-independent prescriptions. On the one hand, one of the most well-known examples of aggregation artifact is that of learning curves (Estes, 1956; Gallistel et al., 2004; Sidman, 1960). As has long been recognized, averaging a family of discontinuous learning curves of the kind produced by insight-based, all-or-none learning in which the insight point occurs at different times can produce a smoothly increasing group curve of the kind predicted by incremental-learning, linear operator models (Batchelder, 1975). That is, the conclusions one would draw at the group and individual levels are diametrically opposed. Zealously averaging over unknown individual differences can produce results that potentially misdirect the theoretical direction of the entire field. For example, Liew et al., (2016) recently demonstrated that attempts to model several types of context effects, such as the similarity, attraction, and compromise effects which arise in multiattribute decision-making, simultaneously obscures the fact that these effects do not appear concurrently in any single individual but only in aggregate.

On the other hand, studies of speeded decision-making are often carried out on group data created by averaging quantiles of response time distributions across participants (Ratcliff & Smith, 2004). Fits of the diffusion decision model to quantile-averaged group data typically agree fairly well the averages of fits to individual participant data (Ratcliff et al., 2003; Ratcliff et al., 2004). The reason is because of the affine quantile structure of many empirical families of response time distributions, which is captured by the diffusion model (Ratcliff & McKoon, 2008; Smith, 2016), and which allows quantile-averaging of distributions without distortion (Thomas & Ross, 1980).^3^ The empirical affine structure is likely to be only approximate rather than exact (Rouder et al., 2010)–which is why individual-level verification is always advisable—but is often enough that the group- and individual-level pictures turn out to be in good agreement. In these situations, the value of averaging is primarily the pragmatic one of data smoothing, rather than the inferential one of estimating population parameters.

Similar results on the effects of aggregation were reported in a number of other cognitive tasks by Cohen, Sanborn, and Shiffrin (2008). They investigated models of forgetting, categorization, and information integration and compared the accuracy of parameter recovery by model selection from group and individual data. They found that when there were only a small number of trials per participant parameter recovery from group data was often better than from individual data. Like the response time studies, their findings demonstrate the data-smoothing properties of averaging and the fact that smoother data typically yield better parameter estimates. In the case of response time studies, which usually collect large samples of data from individual participants, the benefits of aggregation arise mainly in relation to estimating model parameters that depend on parts of the data space that are sampled only sparsely, such as the tails of distributions of error responses to highly discriminable stimuli. Cohen et al.’s results also highlight the fact that, while distortion due to aggregation remains a theoretical possibility, there are no universal prescriptions about whether or not to aggregate; aggregation artifacts must be assessed on a case-by-case basis rather than asserted or denied a priori.

From an inference perspective, the practice of fitting models at the individual participant level has one particularly profound consequence, namely, *the individual, rather than the group, becomes the replication unit.* That is, a small-*N* design that reports measurements and model fits for, say, three, six, or ten participants is actually reporting three, six, or ten independent replications of the experiment. As J. Ross (2009) noted, the high degree of measurement precision afforded by the use of psychophysical methods in vision science means there is often a high degree of uniformity in measurements and model fits across participants. From this perspective, an article that reports a consistent set of measurements and fits across three participants is not statistically underpowered; rather, it is doing what the OSC has charged that cognitive and social psychology typically fail to do and carrying out multiple replications. From this perspective, the goal of “replication” is not to estimate population parameters; it is to ascertain whether the same functional relationships and the psychological processes they reflect are exhibited consistently across individuals. We believe that the reason why vision science and related areas are apparently not in the grip of a replication crisis is because of the inbuilt replication property of the small-*N* design. This property, combined with psychophysical measurement methods that produce a high degree of consistency across individuals, means that many published papers in vision science serve as their own replication studies.

As we emphasized earlier, we are not attempting to claim that population-level inferences are unimportant. In studies of individual differences, or in studies of special participant populations, inferences about population parameters are evidently central. However, questions about population structure, and the population parameters that characterize them, are arguably better approached using more refined techniques for characterizing populations than simple averaging, such as the methods developed by Lee and colleagues (Bartlema et al., 2014; Lee & Wagenmakers, 2005), which seek to discover the best latent population structure underlying a set of data empirically. From this perspective, the small-*N* and large-*N* are not mutually exclusively approaches to doing inference; rather they are ends of a continuum. Processes that are conceptualized theoretically at the individual level are best investigated using designs that allow tests at the individual level, which leads most naturally to the small-*N* design. Genuine questions about the distributions of those processes within populations—as distinct from the vaguely defined populations that are invoked in standard inferential statistical methods—naturally lead to larger-sample designs, which allow the properties of those populations to be characterized with precision. As emphasized by Meehl (1967), the style of research that remains most problematic for scientific psychology is research that is focused on demonstrating the existence of some phenomenon, as distinct from characterizing the processes and conditions that give rise to and control it. We illustrate the distinction, with examples, below.

## Phenomenon-based versus process-based research

Consider the following two research questions: The first question, taken from Cohen’s (1990) paper questioning psychological research practices, tests whether children from poorer socioeconomic backgrounds perceive the sizes of coins to be larger than estimates provided by children of wealthier socioeconomic background. The second question, reported in a conference paper presented at Fechner Day by Helen Ross (1990), tests whether participants who grew up and live in rural settings are more susceptible to horizontal line illusions like the Mueller-Lyer illusion than are participants who grew up and live in urban settings. (H. Ross also evaluated the converse hypothesis that participants from urban environments are more susceptible to vertical line illusions than are participants from rural settings.) On the surface, these two questions seem similar to each other, and, indeed, both involve weak, ordinal predictions about the direction of the statistical test. However, only the second of these hypotheses is based on theorizing about the underlying *process* that could conceivably lead to a difference in illusion susceptibility. The general hypothesis is that the distribution of orientation-tuned neurons in the visual system varies as a function of the distribution of orientations in the visual environment (Coppola et al., 1998; Switkes et al., 1978). The structure of the environment therefore has a direct influence on perception which in turn affects judgments based on spatial aspects of the environment (cf. Furmanski and Engel (2000)). While attempts to directly replicate Ross’s original results, which supported the proposed hypothesis, have not to our knowledge been conducted, similar results have been reported by others (Segall et al., 1963).

By contrast, the first question does not have any strong link to theories of perception. Instead, the logic seems to be that there might be some high-level conceptual influence resulting from values or experience that leads to a change in the perception of size. The key point is that the processes which lie between activation of the concept and influence of perception are not specified with any detail. The aim of asking such a question is therefore not to elucidate any theoretical prediction but instead, to demonstrate some *phenomenon* that would presumably prompt radical revision of hypotheses about the interaction of concepts and perception. Cohen’s hypothetical study has in fact been carried out numerous times, apparently first by Bruner and Goodman (1947) who found that poor children do overestimate coins sizes compared to rich children *even when the coins are physically present as a comparison*! In a serendipitous demonstration of cyclical trends in psychological science, this experiment was soon repeated with the result failing to replicate (Carter & Schooler, 1949) and in some cases reversing (Rosenthal, 1968).^4^ This type of atheoretical effects-driven research acts as proxy for the entire category of research focused on demonstrating phenomena rather than examining process. The problems with this approach are further evinced, to take one example, by the recent history of research on social priming starting from Kahneman’s (Kahneman 2011, p. 57) statement that “disbelief...[in social priming results]...is not an option” to a series of high profile replication failures (Doyen et al., 2012; Pashler et al., 2012; Rohrer et al., 2015). Changing the focus from whether or not some behavior occurs to the process underlying it allows a progressively more refined picture to be built up of the conditions under which the behavior occurs and the likely mechanisms that give rise to it. In the remainder of the article, we argue that adopting a small-*N* approach to testing process-based predictions allows for much stronger inferences than the corresponding large-*N* approach.

## Individual- versus group-level inference: a simulation using additive factors

In this section, we illustrate the difference between individual- and group-level inference in order to highlight the superior diagnostic information available in analyzing individuals in a small-*N* design and the problems of averaging over qualitatively different individual performance in a group-level analysis. For this exercise, we have chosen to use Sternberg’s additive factors method (Sternberg, 1969). The additive factors method has been highly influential in the cognitive literature as a method for characterizing the structure of the processes underlying response time (a Google Scholar search reveals more than 3700 articles that reference it) and has been the catalyst for much later theorizing about mental architecture (McClelland 1979; Schweickert 1978, 1980; Townsend & Nozawa 1995). Our primary reason for using the additive factors method is that it occupies something of a middle ground between the kinds of strong mathematical models we emphasized in the preceding sections and the null-hypothesis significance testing approach that was the target of the OSC’s replication study. One likely reason for the historical success of the additive factors method is that it was a proper, nontrivial cognitive model that was simple enough to be testable using the standard statistical methods of the day, namely, repeated-measures factorial analysis of variance. Off-the-shelf repeated-measures ANOVA routines became widely available during the 1970s, the decade after Sternberg proposed his method, resulting in a neat dovetailing of theory and data-analytic practice that undoubtedly contributed to the method’s widespread acceptance and use. By using the additive factors method as a test-bed we can illustrate the effects of model-based inference at the group and individual level in a very simple way while at the same time showing the influence of the kinds of power and sample-size considerations that have been the focus of the recent debate about replicability.

The additive factors method originated as a way to determine the presence or not of sequential or serial stages of processing in response time (RT) tasks (Sternberg, 1969). Two factors, varied factorially, which influence different processes, will have additive effects on response time under the assumption that the processes are arrayed sequentially. The additivity of two sequential stages can be assessed by examining the significance of the interaction effect in a 2 × 2 ANOVA. If the interaction is significant, then the influence of the two factors on RT is not additive, in which case, other processing architectures are inferred (such as the common influence of both factors on a single processing stage). Hence, unlike a *point-null hypothesis*, which arises as a vague prediction of ordinal hypotheses and is almost always false (Meehl, 1967), an interaction effect of 0 is a *point prediction*, which provides for a strong inferential test of the serial model as a model of individual performance.

In the following demonstration, we simulated response times (RTs) from a small number of hypothetical participants in an additive factors design. To characterize the individual-level effects, we bootstrapped each of these participants’ data by downsampling with replacement.^5^ For each bootstrap sample, we conducted both group- and individual-level analysis of the RTs in order to characterize the exact value of the interaction. To characterize the group effects, we averaged the mean RTs from the stimulus conditions for each simulated participant and analyzed the results using a standard repeated-measures ANOVA. Thus, we illustrate the different goals of each method: estimating the value of a parameter, in the case of an individual-level analysis, and inferring whether a population-level interaction is different from the null, in the case of the group-level analysis. The bootstrapping procedure allowed us to establish the variability in the *power* (i.e., the probability of detecting a significant result when one is present) for each method given a fixed data set. We repeated this sampling procedure a number of times to examine the variability in the presence of the effect in our small sample characterizing the power of each method across the range of interaction effect sizes.

In comparing group and individual analyses in this way, we do not intend to imply that this is would be the preferred way to analyze data from an experiment of this kind if such a study were carried out today. For many researchers, the method of choice for analyzing these kinds of data would be hierarchical modeling, using either classical or Bayesian methods (Kruschke, 2014; Rouder et al., 2012; Thiele et al., 2017). Hierarchical methods have several attractive features, particularly when there are only limited data available for each participant (Ratcliff & Childers, 2015); but they do require the researcher to specify a compound model: one level of the model specifies the process that generates the individual-level effects and the other level specifies the population distribution of the parameters of the process. Such models can fail if either of these submodels is misspecified, so inferences about processes at the individual level become conditional on a correctly specified population model. They also yield solutions in which the individual parameters tend to cluster towards to the center of the population distribution, particularly when there are limited individual data, a phenomenon known as “shrinkage.” Moreover, they lack the attractive self-replicating properties of individual analyses in a small-*N* design. Here we have chosen for didactic purposes to report the results of a “bare-hands” analysis to highlight the differences in sensitivity provided by group- and individual-level analysis. The group-level analysis, of average effects using repeated-measures ANOVA, is intentionally the simplest and most common way of characterizing treatment effects in a population, but this is precisely the kind of analysis that has been the focus of the recent debate about replication failure and the associated calls for methodological reform.

### Simulation details

Here we provide an intuitive overview of our simulation; details can be found in the Appendix. For each simulated participant, for each item condition, we sampled 400 RTs from a log-normal distribution. Four hundred trials is a fairly typical sample size for a small-*N* design in which the researcher is attempting to characterize the RT distributions in each condition for each participant individually. We chose the number 400 on this basis rather than on the basis of any formal power analysis. We attach no strong theoretical interpretation to the log-normal and use it simply as a flexible family of unimodal positively skewed distributions that has sometimes been used to characterize distributions of RT empirically (Woodworth & Schlosberg, 1954). The parameters of each item distribution were linked via a linear equation in which the interaction effect was either present or absent.

For the group analyses, we simply averaged the resulting mean RTs for each item across subjects and conducted a 2 × 2 ANOVA. We then repeated this procedure by bootstrapping the sampled RTs from each participant 1000 times to get an estimate of the variability of the ANOVA results. New samples of participants were generated with different proportions of subjects having a null interaction. We drew 20 independent samples with a proportion of [.10,.25,.50,.75,.90] having a null effect. To estimate the power for this analysis, we simply estimated the proportion of bootstrapped samples which had a significant interaction effect (using an *α* level of .05).

For the individual analyses, we used two methods. One method estimated the size of the interaction effect using weighted linear regression. Here we also estimated the power using the proportion of bootstrapped samples which had a significant effect. The other method was to fit a log-normal distribution to the data using maximum likelihood estimation. We fit a general model, in which the interaction term was allowed to be non-zero, and a constrained model, which forced the interaction term to zero. We then used model selection (i.e., a *G*^2^ test) to determine whether the more general model fit significantly better than the constrained model.

### Simulation results

**Fig. 1:**
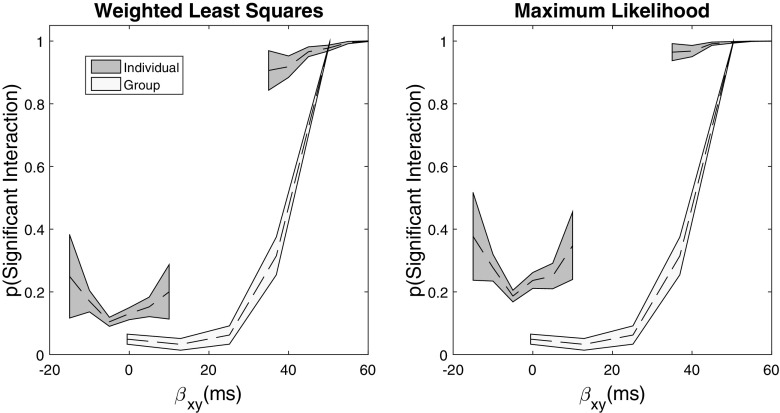
Simulated results for *N* = 4 participants. The dotted line shows the average power estimate, and the shaded region is the bootstrapped 95*%* confidence intervals for each analysis. The *x*-axis is the magnitude of the interaction parameter in the generating model (Equation A4); the *y*-axis is the proportion of times in which a significant interaction was identified in the simulations

In our first simulation, we set *N* = 4. The results of this simulation are shown in Fig. 1. For the individual-level analyses, we have binned the estimated power by the *true* simulation value of the individual interaction effect; for the group analysis, we binned the estimated power by the true average interaction effect for the simulated group. For both analyses, we used a bin size of 5 ms. The results of the weighted least squares analysis are shown on the left panel and the maximum likelihood parameter estimation method are shown on the right. The results of the group-level ANOVA are shown in both panels for comparison. The dotted line presents the average power at the true estimate level, and the shaded patch is the bootstrapped 95*%* confidence intervals.

The results for the weighted least squares analysis and the maximum likelihood method are qualitatively similar, and we make no further distinction between them except to note that the maximum likelihood estimation appears to be more sensitive to small differences from the null interaction. The next thing to note is that the individual analysis picks up the difference in individuals sampled from the null interaction and the positive interaction quite clearly, with the two distinct regions reflecting the separation between individuals. For the individuals sampled with a positive interaction, the individual analysis is very sensitive with the average power greater than .9 even at the lowest levels of the effect. The individual-level analysis is also sensitive even to small effects near zero, from individuals sampled with a null interaction. Effectively, the analysis is sensitive enough to occasionally detect that the interaction effect is different from zero even when that effect is rather small. Lest one think it problematic that the near-zero results are significant some proportion of the time for the individual analysis, recall that the individual analysis provides an estimate of the interaction for each participant. Consequently, one can examine the value of the estimate to determine its importance rather than relying on a null hypothesis test to decide whether it is or is not actually zero.

By contrast, the group analysis is only showing comparable power when all four of the simulated participants show a positive interaction. When any of the participants in the group is sampled from the null interaction effect, the power of the analysis drops substantially (from near 1.0 to .3). The implication is that the group-level analysis is masking the individual differences in the presentation of the interaction. When half or fewer of the participants show the interaction, the group-level analysis only very rarely detects an interaction. It seems wholly undesirable that one could conclude in favor of the null hypothesis when half of one’s sample shows the effect.

### The effect of increasing *N* on the individual- and group-level analyses

**Fig. 2:**
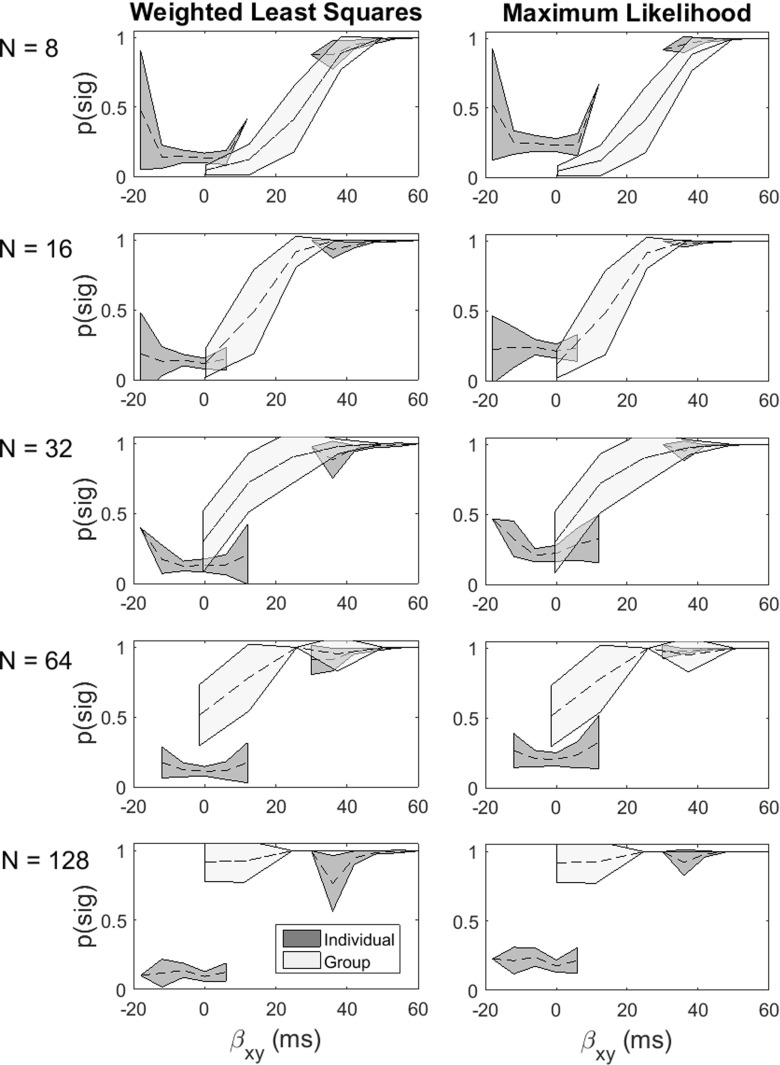
Individual- and group-level analysis as a function of increasing the sample size (from top to bottom). The *x*-axis is the magnitude of the interaction parameter and the *y*-axis is the proportion of significant interactions found in the simulation

We conducted additional simulations in which we increased the sample *N* (see Fig. 2). As *N* increases, the power of the group-level analysis increases as expected, but it is only at large levels of *N* that the group-level analysis is comparable to the individual-level analysis. We would further note that at large *N*, the group-level analysis continues to obscure qualitative individual differences in the level of the effect. Furthermore, the group-level analysis provides no indication of the effect size at the individual level, which, by contrast, the individual-level analysis captures as its primary focus.

## Implications of the simulation study for research practice

There are several important implications of our simulation study that are worth highlighting. First, the tests of the interaction contrast at the individual participant level are carried out with very high power. Statistically, they are tests of the null hypothesis of no interaction; psychologically, they are tests of the cognitive model that the two experimental variables affect different processing stages. The high power is a result of the small-*N* nature of the design: Each participant contributes 400 trials to each of the distributions in the four cells of the *x* × *y* design. These distributions and the precision with which they are estimated determine the accuracy of the estimate of the *β*_*xy*_ parameter and the power of the associated statistical test. Figure 2 shows that these within-participant tests are extremely sensitive to the true value of the parameter. When the true value of the interaction parameter *δ* was 1, the overwhelming majority of tests correctly detected this fact. That is, the tests showed a high degree of replicability, even in the smallest (*N* = 4) design. When the true value of *δ* was zero, and the mean of *β*_*xy*_ was likewise zero, the overwhelming majority of the tests again reflected this fact, but they were also very sensitive to deviations in the sampled value of *β*_*xy*_ from its population mean.

In addition to providing high-powered tests of the interaction at the individual participant level, the tests also provide valuable information about the distribution of *β*_*xy*_ within the population. This information is completely obscured by the group-level analysis, which assumes, a priori, that the effects are sampled from a homogeneous population. The picture that emerges from the group analysis—which is false in this particular instance—is of an experimental design that is substantially underpowered at all but the largest (*N* = 128) sample size. The editorial stance recently adopted by some leading cognitive journals is that large-*N* studies, supported by formal power calculations, are the only way to satisfactorily address questions of this kind. Our simulation highlights just how misleading such an approach can be.

It could be objected that the assumption we made in setting up our simulation study, of a bimodally distributed interaction parameter, was an artificial one, and that anomalies of this kind would be detected by routine data screening. However, it is important to recognize that the bimodality is not at the level of the data but at the level of the parameters of the cognitive model that generated the data. How that bimodality is expressed at the level of the data will depend on a transformation (usually nonlinear, e.g., Eqs. A2 and A3) that expresses the value of the parameter in the observed data. The qualitative expression of bimodality at the level of empirical measurement is merely that some participants display convincing evidence of an interaction while others display weak or no evidence.

In situations of this kind, the logic of small-*N* and large-*N* scientific discovery will unfold very differently. The large-*N* approach would be to run a big sample of participants who, because of resourcing constraints, are likely at best to be minimally practiced on the task and, consequently, highly variable. The large-*N* researcher will view the magnitude of the interaction effect through the lens of the between-participants error variance (repeated-measures ANOVA tests the *x* × *y* interaction against the Participants × *x* × *y* residual term) and will conclude, perhaps based on the results of previous studies, that the treatment effect is somewhat on the small side and will need a large sample to demonstrate reliably. A less well-informed researcher will adopt Cohen’s (1988) definition of “small,” “medium,” and “large” effect sizes as armor against ignorance and likely decide that a “medium” effect size sounds reasonable aspirationally (unlikely to offend journal reviewers), and come to a similar conclusion — although probably on the basis of a different *N*. After a few iterations, the field as a whole will conclude that the effect is somewhat fragile, requires large resources to demonstrate reliably, and is therefore uninteresting theoretically, and will move on to study something else.

The small-*N* approach is somewhat different. The small-*N* design has the advantage of putting the statistical power where it is required theoretically, in the test of the *x* × *y* interaction at the individual participant level. Depending on resourcing and other considerations, the small-*N* researcher might choose to run anywhere between four and a dozen participants. Because the hypothesis tests are carried out at the individual participant level, each participant becomes an independent replication of the experiment and the number of participants who do and who do not show an interaction becomes a rough but useful guide to the replicability of the result. An experimenter who runs 12 participants and finds that ten of them show an interaction can reasonably claim to have identified a fairly, although not perfectly, replicable phenomenon, but one which would still likely merit the attention of colleagues. In this instance, the number of participants showing an interaction could be declared “significant” by a binomial test, using the test in a meta-analytic way to aggregate the results of independent experiments.

An experimenter who runs a small number of participants probably does so in the expectation of finding a high degree of interparticipant agreement, as is often found in sensory science, animal learning studies, and some areas of cognitive neuroscience. However, in situations like the one in our simulation in which there is appreciable heterogeneity in the underlying population, the expected consistency is unlikely to eventuate, or not completely. If, for example, the study used four participants and one or two of them failed to show an interaction, then the experimenter would be forced to acknowledge that the phenomenon, although it may be a real one, is influenced by individual differences whose nature is not properly understood. Nevertheless, the study would have provided useful evidence about the replicability of the finding at the level at which is theorized, namely, at the individual participant level, which would not have been provided by a large-*N* study.

At this point, a number of things could occur. In the absence of fresh insights about the cause of the individual differences, the area might simply stagnate. This occurred, briefly, at the end of 1970s with the additive learning models of criterion-setting in signal detection (Dorfman & Biderman, 1971; Kac, 1962; Thomas, 1973). During the 1970s, the one of the goals of research in this area was to identify models of criterion-setting that predict probability matching in psychophysical decision tasks (the relative frequency of signal responses made by the participant matches the relative frequency of signal stimuli presented by the experimenter). An influential small-*N* study using 12 participants by Dusoir (1980) showed that participants, individually, did not uniformly match, overmatch, or undermatch, and that none of several candidate models under consideration could characterize the performance of all participants. While the immediate effects of this study were negative and led to a pause in this line of research, the end result was not permanent stagnation, but the development of more sophisticated models that were better able to capture the fine-grained structure of sequential effects in decision-making (Treisman & Williams, 1984). These developments served as antecedents for work on sequential effects in decision-making that continues actively up to the present (Jones et al., 2013; Laming, 2014; Little et al., 2017).

The second thing that might occur is that the individual differences could become a research question in their own right. As discussed earlier, sophisticated methods for investi gating these kinds of questions now exist. Lee and colleagues (Lee & Webb, 2005; Bartlema et al., 2014) have shown how cognitive models of individual task performance and population models of the distributions of individual-level parameters can be combined and jointly estimated using hierarchical modeling techniques. Their approach allows researchers to investigate in a principled way whether distributions of model parameters are better characterized as coming from a single population with between-participant variability or from multiple populations. Lee and colleagues use hierarchical Bayesian methods to develop their models, but latent-class mixture models of population heterogeneity can also be investigated using classical (maximum likelihood) statistical methods using the EM (expectation maximization) algorithm (Liang & Bentler, 2004; Van der Heijden et al., 1996). Unlike the automatic use of such samples in large-*N* designs, however, the use of larger samples in these circumstances arises from the systematic attempt to characterize individual differences that were initially identified in small-*N* studies and which would have remained more or less invisible if viewed through a large-*N* lens.

Our example of an additive factors study with a bimodally distributed interaction parameter was a hypothetical one, intended to illuminate the relationship between small-*N* and large-*N* designs, but it is nevertheless interesting to reflect on what would be the implications for scientific inference of a result like the one in Fig. 1—that some participants demonstrate an interaction whereas others do not—should it have been obtained experimentally. The additive factors method purports to aid in identifying the processing architecture of the cognitive system, which would be expected to be stable across individuals, so the existence of individual differences in the interaction would call this inference into question. One possibility might be that there are individual differences in the effectiveness of the experimental manipulations of Factors *x* and *y*. However, in applications of the additive factors method the *x* × *y* interaction term is usually only tested for significance once the main effects of *x* and *y* have both been shown to be significant. The tests of the main effects serve as checks that the experimental manipulations of *x* and *y* separately affect response time, and the test of the interaction is theoretically meaningful only once this has been shown to be the case. We omitted the tests of the main effects from the report of our simulation study but, following the logic of small-*N* designs, they would ordinarily be performed at the individual participant level to provide a manipulation check for each participant individually.

If significant differences among participants in the interaction were found under these circumstances, this in itself, irrespective of any other experimental finding, would probably lead researchers to investigate other models of the cognitive architecture. Other models of cognitive architecture, such as McClelland’s (1979) cascade model and Schweickert’s (1978, 1980) PERT networks, relaxed the restrictive assumptions made by the additive factors method and can predict a richer variety of interactions that might be more consistent with significant individual differences. Sternberg’s (1969) model assumes serial, stage-dependent processing; that is, processing at any stage only commences once its predecessor has finished. McClelland’s model relaxes the stage-dependent processing assumption, and allows partial activation to flow across processing stages, leading to a more general class of *continuous flow* models (Ashby, 1982; Heath, 1992; Sanders, 1990; Miller 1988; Schweickert & Mounts, 1998, Smith, 1995, Smith & Ratcliff, 2009). Schweickert’s PERT network models relax the assumption of serially organized stages and allow for the possibility that the processing system could be comprised of a network of stages, some of which are arranged in parallel and some in series. In these models, two experimental factors affecting different processing stages have additive effects on response times only if the stages both lie on a *critical path* through the network, that is, a sequence of stages, the sum of whose durations determines the overall response time. Otherwise, interactions of some kind are predicted, even when two factors affect different stages. Because the occurrence, sign, and magnitude of such interactions depend on the durations of all of the stages comprising the network, interactions are more common and individual differences in interaction become more plausible than they are in a pure additive factors framework.

This example is a hypothetical one, based on extrapolating from the results of our simulation study as if they were real data, but we offer it in order to illustrate that individual differences in experimental effects need not be inimical to scientific inference. This is especially so when those effects are tested with high power and the associated effect parameters are estimated with high precision. Rather, as the example illustrates, the individual differences themselves give us a strong hint about the kind of explanation we should be seeking — namely, a psychological process or mechanism that is somewhat flexible or malleable, and not an invariant feature of the underlying cognitive architecture. The corresponding large-*N* design provides no such guidance.

Our simulation study has served to highlight one specific advantage of the small-*N* design: the ability to test experimental effects with high power at the individual participant level. However, as our earlier discussion emphasized, in applications these advantages are often accompanied by other features of good scientific practice, namely, strong measurement and the use of explicit mathematical or computational models. In the case of cognitive architectures and the question of serial versus parallel processing, there exist strong inference methods for investigating these questions, and related questions about capacity limitations and exhaustive versus self-terminating processing, based on *systems factorial technology* (Townsend & Nozawa, 1995; Little et al., 2017). Systems-factorial methods identify distinct qualitative signatures of serial and parallel processing, limited-capacity and unlimited-capacity processing, and exhaustive and self-terminating processing, which are expressed empirically at the response time distribution level. Because these inferences are at the level of the distribution, the associated inferences are stronger than are inferences about the means (Sternberg, 1969; Townsend, 1990). These qualitative tests can be augmented by significance tests, which are also carried out at the distribution level (Houpt & Townsend 2010, 2012). As with our simulation study, the scientific logic of these tests is such that they make most sense when applied to individual participants in a small-*N* design.

## Conclusions

The replication crisis marks a crossroads for scientific psychology, and one that is likely to lead to a change in how the discipline carries out its work in future. Some of the recent recommendations for remedying the crisis are based on the premise that we should continue to cleave to the large-*N* design, but beef it up with larger samples and more stringent thresholds for discovery. These proposals, although they are not unreasonable, may nevertheless have fairly severe negative implications for the health of the discipline as whole. Collectively, the discipline has finite resources for running experiments and if these kinds of recommendations become mandated research practice, then they are likely to result in fewer, larger experiments being carried out, fewer research questions being investigated, and an unavoidable impoverishment of psychological knowledge. In view of our finite individual and collective resources for collecting data, we suggest that small-*N* designs are often a better and more informative way to allocate them.

In proposing this, we are not arguing that small-*N* designs are appropriate for every situation. Some of the methods we have discussed in the preceding paragraphs are tools that were developed specifically for the analysis of cognitive architectures and are not applicable to other research areas. We have discussed them in order to illustrate the power that these kinds of methods offer when they are available. Collectively, these methods represent an established and sophisticated way of doing scientific psychology that is very different in style and substance to the one targeted by the OSC’s replication study and characterized as inherently flawed by the scientific and popular press. When the goal is to estimate population parameters, when the phenomenon of interest is highly reactive (i.e., changed by observation), and when only limited data can be collected from individuals, then the recommendation to increase sample size *at the participant level* is an appropriate one.

As we noted earlier, many researchers, particularly in cognitive and mathematical psychology, now favor hierarchical models as providing the best compromise between the number of participants and the number of observations per participant — although as we noted earlier, effective use of such models requires careful specification of population-level submodels. While we fully understand the arguments in favor of such models, to us, many of the published examples of their use have tended to obscure rather than to emphasize the quality of the fits at the individual level. However, our ultimate goal throughout this article is not to criticize these or any other particular methods, but to highlight that psychology is not a homogeneous discipline. In environments that can be explored at the individual level and when the phenomenon of interest is expressed as an individual-level mechanism, small-*N* studies have enormous inferential power and are preferable to group-level inference precisely because they place the burden of sampling at the appropriate level, that of the individual. The lesson is that a common feature of small-*N* methods, and the increased power and precision of inference they offer, is only realizable in *data-rich* environments. It is much more difficult to develop effective methods of strong inference in sparse environments, in which inference depends on significance tests of point hypotheses about means in one or two conditions. In sparse environment, increasing the number participants allows for more precise estimation of the between-participants error terms used to test hypotheses and reduces the likelihood of type I errors. Conversely, the combination of data-rich environments and the availability of methods that allow strong inference will often lead researchers to prefer small-*N* designs.

## Figures and Tables

**Fig. 3:**
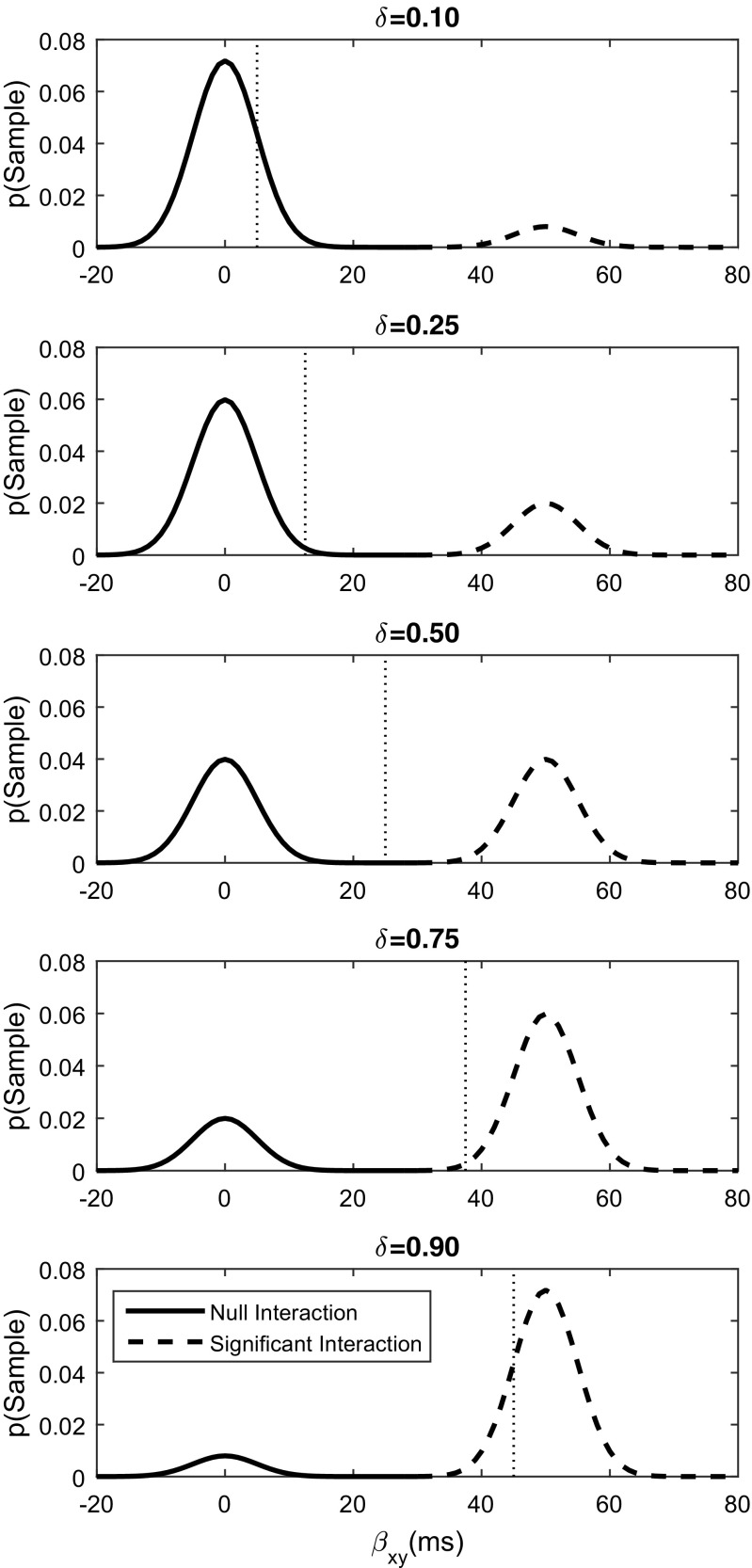
Sampling distributions for each level of *δ*. The *vertical dotted line* indicates the average value for the group. The *panels* show heterogeneous nonnormal populations in which some proportion of the participants exhibit an interaction and some proportion do not. The proportion progressively increases from top to bottom

## Appendix: Details of the additive factors simulation

Consider a 2 × 2 factorial design in which the first factor, *x*, and the second factor, *y*, are varied in two levels. A useful summary statistic, which expresses the cognitive model for the task, is the mean interaction contrast, MIC, defined as the double difference at each level of the factors:

A1 $$\begin{array}{@{}rcl@{}} MIC &=& {\Delta}^{2} E\left( RT\right) = \left[ E\left( RT_{11} \right) - E\left( RT_{12} \right)\right]\\ && - \left[ E\left( RT_{21} \right) - E\left( RT_{22} \right)\right] \end{array} $$

where *E*(*R**T*_*i**j*_) is the mean response time for levels *i* and *j*. This, of course, is the well-known interaction effect in a standard 2 × 2 ANOVA. The theoretical link to processing arises because one can assess whether the MIC is equal to zero, indicating additivity and, hence, serial processing, or different from zero.

RTs for each simulated participant, *i*, were sampled from a log-normal distribution for each factorial level *j* of *x* and *k* of *y* with mean parameter, *μ*_*i**j**k*_, and standard deviation parameter, *σ*_*i**j**k*_ (Gelman et al., 2003). The log-normal parameters were determined by the mean, *m*_*i**j**k*_, and standard deviation, *s*, of the non-log-transformed samples as follows:

A2 $$ \mu_{ijk} = \log\left( \frac{m^{2}_{ijk}}{\sqrt{s^{2} + m^{2}_{ijk}}} \right) $$

A3 $$ \sigma_{ijk} = \sqrt{\log\left( 1 + \frac{s^{2}}{m^{2}_{ijk}} \right)}. $$

The link to the additive factors method is determined by the dependency of *m* on the experimental variables, which is given by the following linear equation:

A4 $$ m_{ijk} =\beta_{0} + \beta_{x} x_{j} + \beta_{y} y_{k} + \beta_{xy} x_{j} y_{k}, $$

where *x*_*j*_ and *y*_*k*_ take on the values 0 and 1 reflecting the stimulus condition (i.e., they function like regression dummy variables). The standard deviation of the log normal distribution was set to *s* = 100 ms. We set the intercept, *β*_0_ equal to 400 ms, and the main effects of *x* and *y* equal to *β*_*x*_ = 150 ms and *β*_*y*_ = 200 ms, respectively. These values were chosen somewhat arbitrarily but produce distributions of RT that resemble those that are found experimentally.

For each simulated participant, we allocated a variable, *δ* ∈ {0, 1}, which determined the presence or absence of an interaction for that participant. If *δ* = 1, then *β*_*x**y*_ was a draw from a normal distribution with a mean of 50 ms and a standard deviation of 5 ms (i.e., an overadditive interaction between *x* and *y*). By contrast, if *δ* = 0, then *β*_*x**y*_ was a draw from a normal distribution with a mean of 0 ms and a standard deviation of 5 ms (i.e., an additive relationship with no interaction between *x* and *y*). The standard deviation for the null effect was non-zero to reflect the potential for error from other sources (e.g., measurement error due to finite sampling of the RTs).

### **Group-Level Analyses**

We first simulated a set of *N* participants with the number of RTs, *n*_*r**t**s*_ equal to 400 in each factorial condition for each factorial condition (i.e., each item) presented to each participant. To generate the RTs, we initially simulated a sample of 500 which was downsampled with replacement to *n*_*r**t**s*_ = 400.

For each bootstrapped set of *N* simulated participants, we averaged the data for each participant in each of the factorial conditions and conducted a 2 × 2 ANOVA. We resimulated the set of *N* participants 100 times allowing *δ* to equal 1 with a probability of [.10,.25,.50,.75,.90]. That is, 20 new simulated data sets were taken at each level of *δ* and then bootstrapped with the individual and group level analyses applied to each bootstrapped sample. Figure 3 shows the sampling distributions for each level of delta. The bootstrapping procedure was repeated 1000 times per set of simulated participants.

### **Individual-Level Analyses**

For each bootstrapped *individual*, we conducted two individual-level analyses. First, we used a weighted general linear model analysis using a normal distribution with an identity link function, and weights for each RT given as $w = \frac {1}{\left [X \left (X \backslash y \right ) \right ]^{2}}$, where *X*∖*y* is the least squares solution of the matrix equation **bX = y**.

For the second analysis, we estimated the parameters of the linear mean equation (see Eq. A4) and arithmetic standard deviation by maximizing the likelihood of the log RTs using a lognormal likelihood function (Gelman et al., 2003):

A5 $$ L\left( \!x_{ijk\cdotp} \right) \,=\, \frac{1}{x_{ijk\cdotp}} \frac{1}{\sqrt{2 \pi \sigma_{ijk}^{2}}} \exp\!\left[\!-\frac{1}{2 \sigma_{ijk}^{2}} \left( \log\left( x_{ijk\cdotp} \right) \,-\, \mu_{ijk} \right)\!\right]\!, $$

where *x*_*i**j**k*⋅_ indicates each individual trial for participant *i* in condition *x*_*j*_ and *y*_*k*_ and *μ*_*i**j**k*_ and *σ*_*i**j**k*_ are given above. We used a Nelder–Mead algorithm (Nelder & Mead, 1965) to minimize the negative log of likelihood in Eq. A5 summed across all of the trials in each of the item conditions. We fit two models: a full model with five parameters, (*β*_0_, *β*_*x*_, *β*_*y*_, *β*_*x**y*_, *s*) and a constrained model in which *β*_*x**y*_ = 0. We then used a nested model comparison test, *G*^2^, to determine the preferred model, where $G^{2} = -2 \left [\log \left (\hat {L}_{\text {full}} \right ) - \log \left (\hat {L}_{\text {constrained}} \right )\right ]$ and $\hat {L}$ is the maximum likelihood estimate (Myung & Pitt, 2004). *G*^2^ is distributed as a *χ*^2^ random variable with degrees of freedom equal to the difference in the number of parameters between the full and constrained model (i.e., *d**f* = 1). Power was estimated for this analysis by counting the number of boostrapped samples which had a significant *G*^2^ value.

## Footnotes

1 It is, of course, also important to realize that there are other sources of variability which are typically uncontrolled and add to the error variance in an experiment. These factors include not only variability across participants but also across the stimuli which are tested, equipment, location, time of day, and so on. Baribault et al., (2018) developed a novel method of determining whether or not any of these factors contributes to changes in a population parameter estimate by randomizing all of the various aspects of the experiment that might contribute to the effect. The specific effect of any factor was then determined by marginalizing over the other factors providing a way to isolate causal variables.

2 The form of statistical inference that most closely corresponds to the form of reasoning we are calling ”Gaussian” is the kind that uses confidence intervals, which have been proposed as an alternative or an adjunct to null-hypothesis testing by various authors (Cumming & Finch, 2005; Estes, 1997; Loftus & Masson, 1994). When used in the context of the kind of large-*N* design that is common in psychology, confidence intervals cannot compensate for the problems of weak measurement and weak theory we have identified here and do not represent a solution to the replication crisis. The power of the approach we are advocating derives from the use of strong theories and computational models that predict entire functions, not just isolated points.

3 “Affine” structure is structure that is more commonly referred to as “linear.” It means that the *j* th quantile for the *i* th observer, *Q*_*i*, *j*_, is linearly related to the corresponding quantile for the *k* th observer, *Q*_*k*, *j*_, by a function of the form *Q*_*i*, *j*_ = *a* + *b* *Q*_*k*, *j*_,where *a* and *b* are constants that depend on the two observers being compared.

4 Bruner and Goodman (1947) also demonstrated greater overestimation by poor children compared to rich children when the estimation was based on memory. This result held in Carter and Schooler’s (1949) replication but reversed in older children in Rosenthal’s (1968) replication. Rosenthal explained his results by positing that familiarity likely played a critical role in estimated from memory. We direct the reader to Firestone and Scholl’s (2016) review of the larger topic of top-down influences on perception.

5 Downsampling (of a larger sample) is typically used in bootstrapping when comparing two samples of unequal size (Racine et al., 2014); here, we applied downsampling to increase the variability of the individual distributions.
